# A novel and highly efficient esterification process using triphenylphosphine oxide with oxalyl chloride

**DOI:** 10.1098/rsos.171988

**Published:** 2018-02-21

**Authors:** Mingzhu Jia, Lixue Jiang, Fanfan Niu, Yu Zhang, Xiaoling Sun

**Affiliations:** School of Chemical and Environmental Engineering, Shanghai Institute of Technology, 201418, Shanghai, People's Republic of China

**Keywords:** triphenylphosphine oxide, coupling reagent, oxalyl chloride, esterification

## Abstract

Triphenylphosphine oxide (TPPO) and oxalyl chloride ((COCl)_2_) are used as novel and high-efficiency coupling reagents for the esterification of alcohols with carboxylic acids via the TPPO/(COCl)_2_ system at room temperature for 1 h. The reaction represents the first TPPO-promoted esterification under mild and neutral conditions with excellent yields. Furthermore, we proposed a plausible mechanism with the help of ^31^P NMR spectroscopy.

## 1. Introduction

**Scheme 1.**
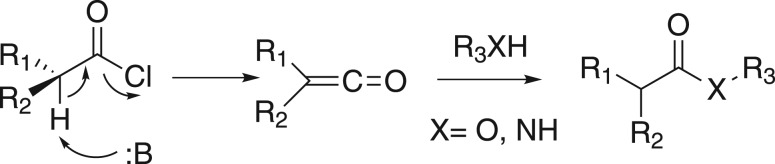
Racemization through the ketene intermediate is a common problem associated with the use of acyl chlorides as reagents in ester (and amide) coupling reactions.

As is well known, carboxylic esters are fundamental organic compounds in organic synthesis and have been widely used in chemical and pharmaceutical industries, such as spices, daily chemical industries, foods, medicines, rubbers, coating materials and so on [1]. Owing to the importance of esters, numerous chemical methods have been reported to accomplish this basic transformation [2,3]. Esters are primarily prepared from the condensation of carboxylic acids with alcohols; generally, the most common methods for the preparation of ester proceed via carboxyl group activation and subsequent reaction with a suitable alcohol [4]. Among them, acid halides were recognized as powerful esterifying agents because of their complete conversion and high yields; however, to the best of our knowledge, acid halides always generate highly acidic by-products such as hydrochloric acid, which could result in decomposition of the initial materials; this method has almost no application in the synthesis of a natural product because of greater possibility of reaction with some acid-sensitive functional groups [2–4]. Moreover, acid chlorides are prone to hydrolysis under basic conditions through the standard ketene intermediate (scheme 1) [4]. Therefore, it is crucial to find a mild coupling system for the further development of chemistry.

Phosphonium, phosphinic salts and phosphine oxides as frequently used coupling reagents have been reported especially for some famous reactions such as the Wittig reaction [5–10], Appel reaction [11–14], Staudinger reaction [15–20] and Mitsunobu reaction [2,21–32]. The Mitsunobu reaction, first reported by Mitsunobu *et al.* in 1967 [21], converts an alcohol into a variety of other functional groups including esters, and this method could generate esters in excellent yield (90%) via the condensation of a carboxylic acid and alcohol with a mixture of triphenylphosphine (Ph_3_P) and diethyl azodicarboxylate (DEAD). More specifically, the Mitsunobu reaction is highly stereospecific and selective; therefore, it is appropriate for preparing some products or derivatives with sensitive groups.

In the Mitsunobu reaction, the alcohol was usually activated towards nucleophilic attack from the carboxylic acid, and this activation was achieved by the reaction with a phosphine, typically Ph_3_P, and a dialkyl azodicarboxylate. In recent years, a number of reports have focused on generating other azo dicarboxylates such as diisopropyl azodicarboxylate [2,22], di-2-methoxyethyl azodicarboxylate [23], azodicarbonyl dimorpholide [24], di-*p*-nitrobenzyl azodicarboxylate [25], 5,5′-dimethyl-3,3′-azoisoxazole [26], 4-dimethylaminopyridine [27,28], di-*p*-chlorobenzyl azodicarboxylate [29], DEAD [30], *N*-chlorobenzotriazole [31] and Fe(Pc) [32].

It is worth noting that the production of carboxylic esters using the Mitsunobu reaction will generate triphenylphosphene oxide (TPPO). The difficulty of removing it from the reaction mixture not only makes this method hard to operate, but also limits large-scale applicability of some reactions. Although many reports have been directed towards modifying Ph_3_P to polymer phosphorus compounds [33–37], the preparation of these polymers was not simple and the treatment of TPPO by-products gave rise to a waste of resources. To overcome these drawbacks, most studies focus on the reduction of TPPO to Ph_3_P [38–40]. Mecinovic and co-workers reported an amide reaction mediated by the PPh_3_/CCl_4_ system, and TPPO was reduced to PPh_3_ via this reaction [41]. Consequently, these phosphorus oxides have been used for several catalytic reactions, such as the catalytic Wittig reaction [5–10], catalytic Appel reaction [11–14] and catalytic Staudinger reaction [15–20]. It is easy to find the operating cost so high that it is not suitable for industrial production. Therefore, it is meaningful to recycle this waste product (TPPO) and reassemble a novel system to substitute the PPh_3_/assistant reagents.

**Scheme 2.**
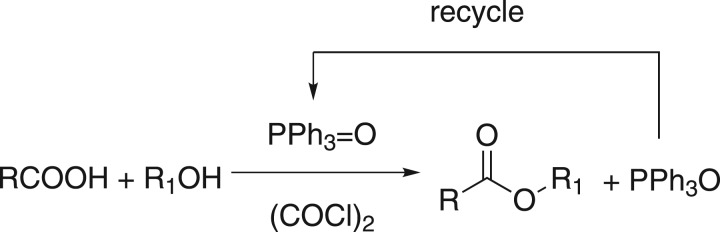
TPPO/(COCl)_2_ applied to esters by using recyclable TPPO.

By searching a large number of works of the relevant literature, it is worth mentioning that the reaction of oxalyl chloride ((COCl)_2_) with TPPO discovered by Fukui & Masaki [42] could form an intermediate (a kind of acyl phosphosphonium salt), and then the potential usefulness of this reaction system for catalytic transformation was exploited by Denton and co-workers in the Appel reaction and other reactions under the Appel conditions [13,14,43–45]. Through the mechanisms of these reactions, we have successfully synthesized amides by using the TPPO/(COCl)_2_ system [46], and hypothesized that this intermediate may be applied for esterification. To our surprise, we found that this acyl phosphonium salt could promote the activation of carboxylic acids first during the experiment and react with alcohols to generate corresponding esters (scheme 2). In this system, TPPO can act as a Lewis base to promote the reaction between acid and alcohol during condensation.

Compared with traditional esterification via acid chloride, our system has the advantages of short reaction time (just 1 h), mild condition (room temperature), excellent yields and high atom efficiency (TPPO can be recycled). Although TPPO increased the complexity of purification, this problem could be ignored with reference to the listed advantages compared with the classical Mitsunobu reaction. This neutral method is applicable for natural product synthesis. Moreover, there is great potential in the chemical industry to resolve the waste due to the by-products of TPPO. Fortunately, there is no report describing the utilization of TPPO with (COCl)_2_ for the esterification system thus far.

## 2. Material and methods

### 2.1. Reagents

CH_3_CN (greater than or equal to 99.0%), CH_2_Cl_2_ (greater than or equal to 99.5%), C_2_H_4_Cl_2_ (greater than or equal to 99.0%), PhMe (greater than or equal to 99.5%), Et_3_N (greater than or equal to 99.0%), TPPO (99%), (COCl)_2_ (98%), PhCOOH (greater than 99.0%), NO_2_-PhCOOH (greater than 99.0%), CH_3_O-PhCOOH (greater than 99.0%), CH_3_-PhCOOH (greater than or equal to 98.0%), Cl-PhCOOH (99.0%), *trans*-cinnamic acid (greater than or equal to 98.0%), picolinic acid (99%), 1-naphthoic acid (greater than or equal to 99.0%), diphenylacetic acid (greater than or equal to 99.0%), PhCH_2_OH (greater than or equal to 99.0%), CH_3_(CH_2_)_3_OH (greater than or equal to 99.0%), CH_3_OH (99.9%), Ph(CH_2_)_2_OH (greater than or equal to 99.0%), 1-adamantanol (99%), CH_3_(CH_2_)_11_OH (greater than or equal to 99.5%) and Ph_2_CH_2_OH (99%).

### 2.2. Methods

#### 2.2.1. Preparation of esters by using TPPO/(COCl)_2_

To a cold solution of TPPO (1.4 g, 5 mmol) in acetonitrile (CH_3_CN, 5 ml), (COCl)_2_ (0.55 ml, 6.5 mmol) was added slowly in drops under magnetic stirring. After 10 min, carboxylic acid (1 equiv, 5 mmol) was added and stirred for 10 min. Then, alcohol (1.3 equiv, 6.5 mmol) and Et_3_N (0.67 ml, 5 mmol) were added in sequence. The reaction was carried out under the protection of nitrogen gas, and the reaction temperature was room temperature. Stirring was continued for 1 h. The progress of reaction was followed by thin layer chromatography (TLC). After the reaction, mixture was evaporated *in vacuo* and the final product was purified by column chromatography with petroleum ether/ethyl acetate (8 : 1) as the eluent. All esters presented in table 3 are previously known and reported compounds.

#### 2.2.2. Procedure for esterification (table 1)

TPPO (1.4 g, 5 mmol), CH_3_CN (5 ml), (COCl)_2_ (0.55 ml, 6.5 mmol), PhCOOH (0.67 g, 5 mmol), PhCH_2_OH (0.67 ml, 6.5 mmol) and Et_3_N (0.67 ml, 5 mmol) were added as in the previous procedure. The reaction was carried out under different reaction temperatures and times. In the end, the yield was measured by TLC (table 1).

**Table 1.** Esterification of benzoic acid and benzyl alcohol by using different organic solvents under different temperatures and reaction times.

entry*^a^*	organic solvent	temperature (°C)	time (h)	isolated yield (%)
1	CH_3_CN	28	1	90
2	CH_3_CN	28	2.5	89
3	CH_3_CN	30	4	86
4	PhMe	30	1	86
5	CH_2_Cl_2_	30	1	89
6	C_2_H_4_Cl_2_	30	1	88
7	CH_3_CN	50	1	90
8	CH_3_CN	70	1	90

*^a^*Reaction conditions: benzoic acid (5 mmol), benzyl alcohol (1.3 × 5 mmol), solvent (5 ml), Et_3_N (5 mmol) under nitrogen.

#### 2.2.3. Procedure for recycling TPPO

At the end of the reaction, the mixture was evaporated *in vacuo* and corresponding esters with TPPO were separated by column chromatography. The residue was purified with an eluent of 8 : 1 petroleum ether/ethyl acetate and then the polarity of the eluent was changed to 2 : 1, and TPPO was obtained. The crude product was evaporated and dried *in vacuo*. The white solid obtained was the reusable TPPO.

## 3. Results and discussion

As mentioned above, in order to optimize the reaction conditions, we firstly chose benzoic acid (1 equiv) and benzyl alcohol (1.3 equiv) as reaction substrates, which were stirred in CH_3_CN for 1 h at room temperature under the protection of nitrogen gas and then was added TPPO (1 equiv)/(COCl)_2_ (1.3 equiv); the reaction mixture was neutralized by triethylamine (Et_3_N), and the desired ester, benzyl benzoate, was obtained in a 90% yield (table 1, entry 1). Initially, we tested the influence of reaction time from 1 to 4 h and different organic solvents including CH_3_CN, PhMe, CH_2_Cl_2_ and C_2_H_4_Cl_2_ at different temperatures to find the most suitable conditions for the reaction; the best results are summarized in table 1.

According to the results obtained in different organic solvents (table 1, entries 4–6), the esterification yields had no obvious difference. As is well known, TPPO has a better solubility in CH_2_Cl_2_ or C_2_H_4_Cl_2_ than CH_3_CN and PhMe, and during the experimental phenomena, we found that the solid in CH_3_CN was dissolved rapidly after the addition of (COCl)_2_, and there were no obvious changes in the phenomena in other solvents; hence we chose CH_3_CN as the reaction solvent.

Moreover, by monitoring the reaction using TLC, we found that the reactant was consumed within 1 h and that extended reaction time cannot improve product yields appreciably. By contrast, as the reaction progressed, some generated esters were decomposed because of the reversibility of the reaction (table 1, entries 1–3). Generally, temperature is an important factor for various reactions. However, it had no obvious influence in this system as seen in table 1, entries 6–8.

**Scheme 3.**
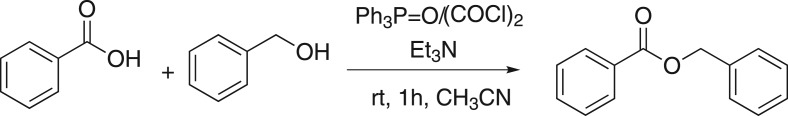
Reaction giving benzyl benzoate in the presence of TPPO with (COCl)_2_.

We also examined the effect of different ratios of TPPO/(COCl)_2_/PhCOOH/PhCH_2_OH/Et_3_N in CH_3_CN at room temperature for the conversion to benzyl benzoate (scheme 3); the results are summarized in table 2.

**Table 2.** Esterification with different ratios of TPPO/(COCl)_2_/PhCOOH/PhCH_2_OH/Et_3_N in CH_3_CN at room temperature.

entry*^a^*	molar ratio TPPO/(COCl)_2_/R_1_COOH/ROH/Et_3_N	isolated yield / %
1	1/1/1/1.3/1	79
2	1/1.3/1/1.3/1	90
3	1/2/1/1.3/1	88
4	1/0.75/1/1.3/1	70
5	0.75/1.3/1/1.3/1	50
6	2/1.3/1/1.3/1	89
7	1/1.3/1/1/1	68
8	1/1.3/1/2/1	90
9	1/1.3/1/1.3/1.3	90
10	1/1.3/1/1.3/2	90
11	1/1.3/1/1.3/0.75	85
12	1/0/1/1.3/1	nr
13	0/1.3/1/1.3/1	nr

*^a^*Reaction conditions: solvent (5 ml), room temperature, 1 h, under nitrogen.

As the esterification reaction of benzoic acid to benzyl benzoate gave a 90% yield (table 3, entry 1), we first applied our reaction conditions to the carboxylic acids carrying both electron-donating groups (--OMe and --Me) (table 3, entries 3 and 4) and electron-withdrawing groups (--NO_2_ and --Cl) (table 3, entries 2, 5 and 17), and these carboxylic acids gave their corresponding esters in good yields. However, there was a little difference between electron-donating groups and electrophilic groups. The position of substituent groups could affect the esterification yields (table 3, entries 2 and 17); it was easy to find that *m*-nitrobenzoic acid has lower conversion (90%) compared to *p*-nitrobenzoic acid (94%). By now, we clearly realized that the double bond had a crucial role in the esterification (table 3, entry 6); it improved the yield (95%). Of course, steric effects via acids were examined. It was evident that the steric hindrance of acids is an influencing factor for the esterification yield (table 3, entries 7, 8 and 16), which reduces the conversion appreciably.

**Table 3.** Condensation of carboxylic acids with different alcohols in the presence of the TPPO/(COCl)_2_/Et_3_N system.

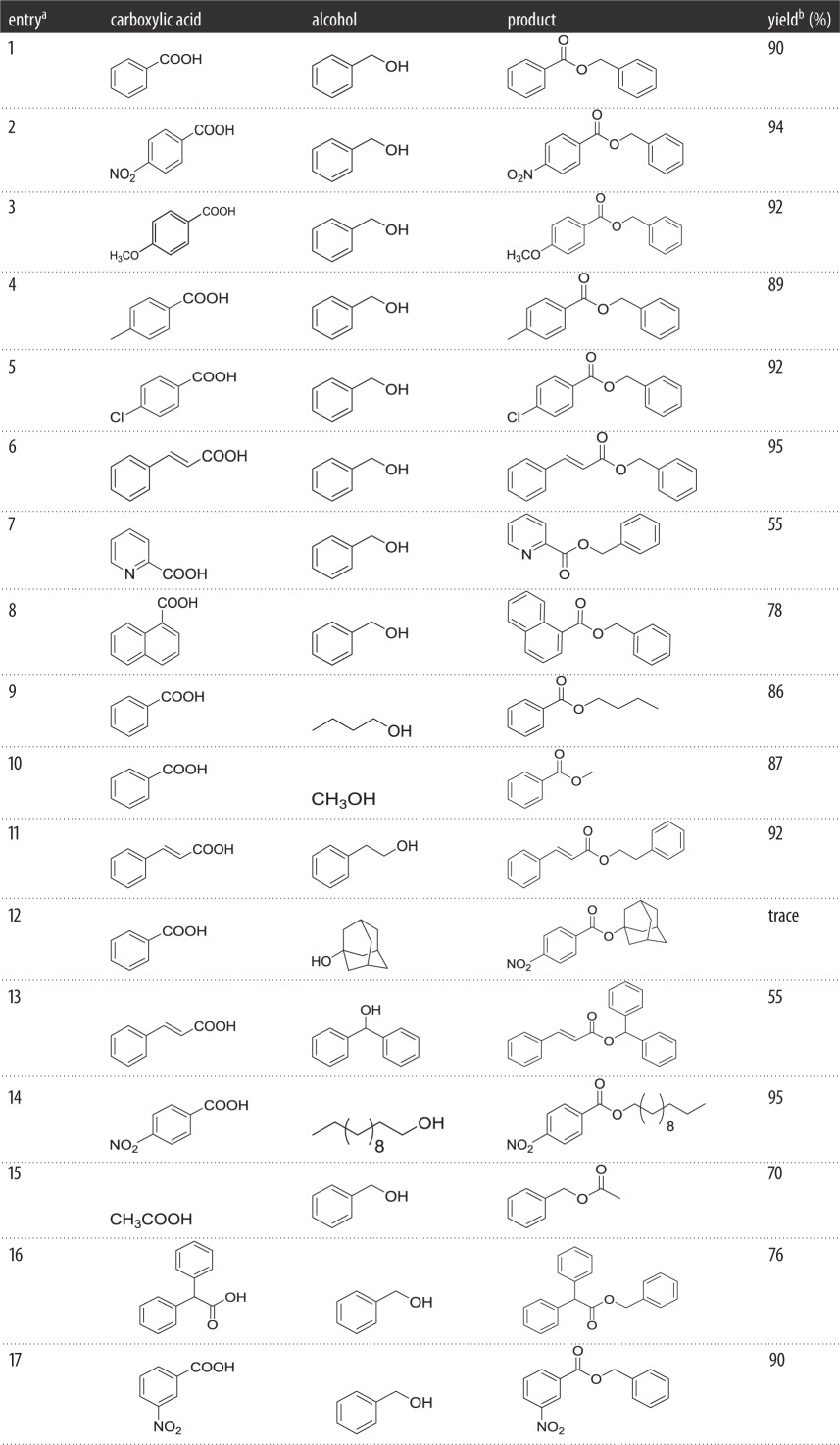

*^a^*Reactions were carried out with RCOOH (5 mmol, 1 equiv), R_1_OH (6.5 mmol, 1.3 equiv), TPPO (5 mmol, 1 equiv), (COCl)_2_ (6.5 mmol, 1.3 equiv) and Et_3_N (5 mmol, 1 equiv) in CH_3_CN (5 mL) at room temperature for 1 h.

*^b^*Isolated yield.

In comparison, aliphatic alcohols had lower reactivity than aromatic alcohols (table 3, entries 1 and 9, 1 and 10). However, as is to be expected, this impact could be ignored when employing electron-deficient carboxylic acids (table 3, entries 6 and 11, entries 2 and 14). Moreover, the aromatic acid was more reactive than the aliphatic acid (table 3, entries 1 and 15) just as we expected. Tertiary alcohols because of their steric hindrance were tested to react with benzoic acid; as we expected, there was little corresponding ester generated (table 3, entry 12); besides, secondary alcohols gave lower yields for the same reason (table 3, entries 6 and 13).

To explore the effect between substrates and corresponding yields accurately, we have provided a summary about the times of reaction for different substrates in table 4. The progress of the reaction was followed by TLC, and the final product was purified by column chromatography.

**Table 4:** Optimization of triphenylphosphine oxide promoted esterification.

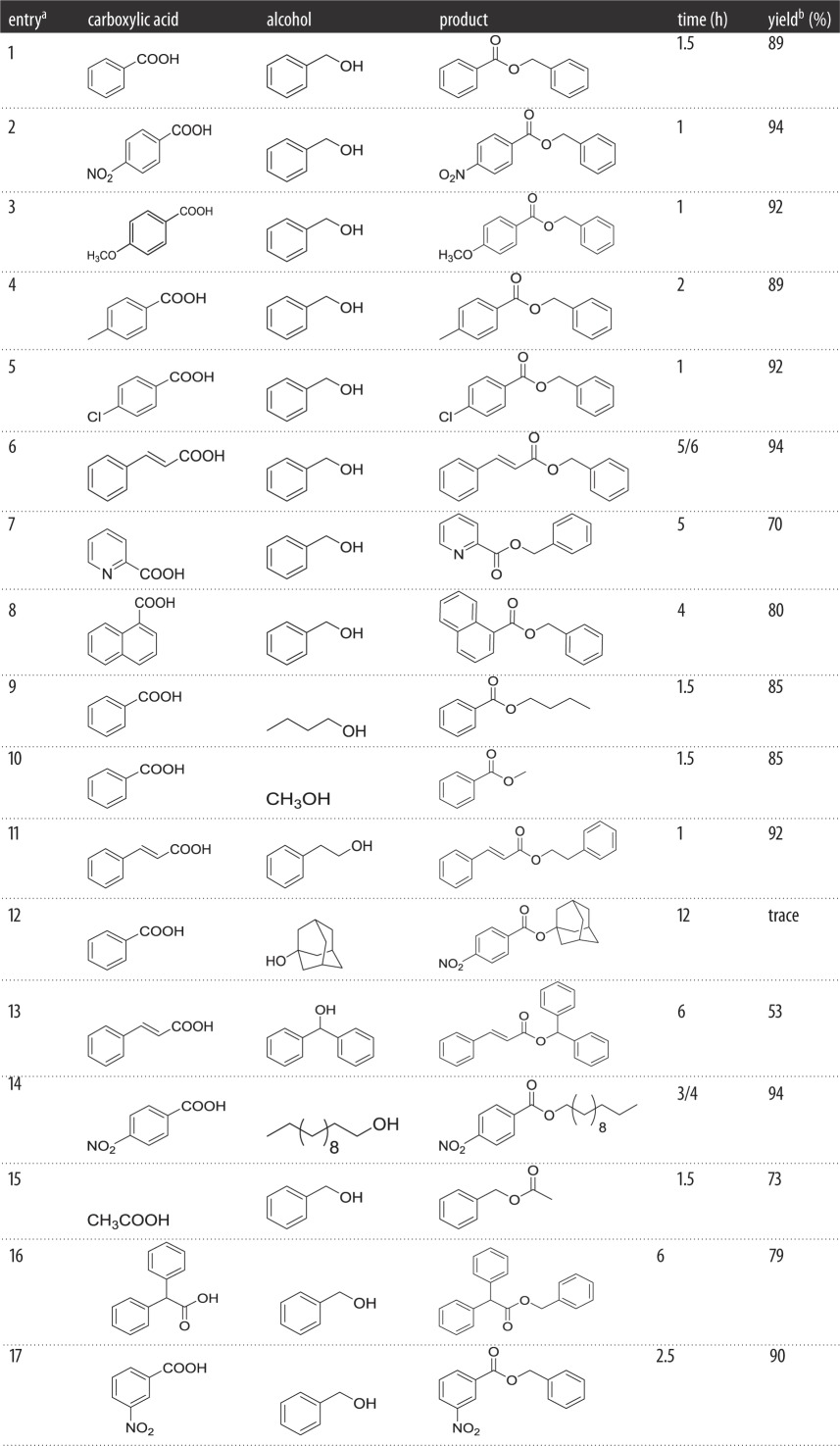

*^a^*Reactions were carried out with RCOOH (5 mmol, 1 equiv), R_1_OH (6.5 mmol, 1.3 equiv), TPPO (5 mmol, 1 equiv), (COCl)_2_ (6.5 mmol, 1.3 equiv) and Et_3_N (5 mmol) in CH_3_CN (5 ml) at room temperature.

*^b^*Isolated yield.

As shown in table 4, some cases indicated that the reactants were consumed at 1 h, and most substrates proved that increased reaction time will not change yields greatly, with the exception of table 4, entry 7 by TLC. In conclusion, esterification was difficult to be carried out for substrates with bulky chemical constitution.

### 3.1. Benzyl benzoate (table 3, entry 1)

^1^H NMR (500 MHz, CDCl_3_) *δ* 8.13 (d, *J* = 7.8 Hz, 2H), 7.59 (t, *J* = 7.4 Hz, 1H), 7.53–7.36 (m, 7H), 5.41 (s, 2H); ^13^C NMR (125 MHz, CDCl_3_) *δ* 165.57, 135.86, 131.14, 128.77, 128.69, 128.64, 128.42, 128.29, 66.98; HRMS (FT ICR-MS) *m/z*: [M + Na]^+^ calcd for C_14_H_12_O_2_ 235.07295; found 235.07294.

### 3.2. Benzyl 4-nitrobenzoate (table 3, entry 2)

^1^H NMR (500 MHz, CDCl_3_) *δ* 8.28 (dd, *J* = 20.8, 8.9 Hz, 4H), 7.56–7.35 (m, 5H), 5.46 (s, 2H); ^13^C NMR (125 MHz, CDCl_3_) *δ* 164.54, 150.62, 135.53, 135.29, 130.84, 128.77, 128.67, 128.45, 123.56, 67.66; MS *m/z*: [M] calcd for C_14_H_11_O_4_N 257.24; found 257.

### 3.3. Benzyl 4-methoxybenzoate (table 3, entry 3)

^1^H NMR (500 MHz, CDCl_3_) *δ* 8.09 (t, *J* = 13.7 Hz, 2H), 7.42 (ddd, *J* = 25.3, 20.3, 7.2 Hz, 5H), 6.96 (t, *J* = 14.7 Hz, 2H), 5.38 (s, 2H), 3.88 (s, 3H); ^13^C NMR (125 MHz, CDCl_3_) *δ* 166.20, 163.48, 136.36, 131.77, 128.59, 128.17, 128.12, 122.59, 113.66, 66.41, 55.43; MS *m/z*: [M + Na]^+^ calcd for C_15_H_14_O_3_ 242.27; found 242.

### 3.4. Benzyl 4-methylbenzoate (table 3, entry 4)

^1^H NMR (500 MHz, CDCl_3_) *δ* 8.00 (d, *J* = 8.0 Hz, 2H), 7.48–7.30 (m, 5H), 7.26 (d, *J* = 7.9 Hz, 2H), 5.39 (s, 2H), 2.43 (s, 3H); ^13^C NMR (125 MHz, CDCl_3_) *δ* 166.52, 143.73, 136.33, 129.81, 129.15, 128.63, 128.22, 128.16, 127.54, 66.53, 21.66; HRMS (FT ICR-MS) *m/z*: [M + Na]^+^ calcd for C_15_H_14_O_2_ 249.08860; found 249.088703.

### 3.5. Benzyl 4-chlorobenzoate (table 3, entry 5)

^1^H NMR (500 MHz, CDCl_3_) *δ* 8.05 (t, *J* = 11.4 Hz, 2H), 7.47 (d, *J* = 6.4 Hz, 2H), 7.45–7.37 (m, 5H), 5.41 (d, *J* = 20.2 Hz, 2H); ^13^C NMR (125 MHz, CDCl_3_) *δ* 165.60, 139.53, 135.81, 131.13, 128.76, 128.67, 128.60, 128.41, 128.28, 66.97; MS *m/z*: [M + Na]^+^ calcd for C_14_H_11_O_2_Cl 246.69; found 246.

### 3.6. Benzyl cinnamate (table 3, entry 6)

^1^H NMR (500 MHz, CDCl_3_) *δ* 7.78 (d, *J* = 16.0 Hz, 1H), 7.56 (dd, *J* = 6.1, 3.0 Hz, 2H), 7.49–7.34 (m, 8H), 6.54 (d, *J* = 16.0 Hz, 1H), 5.30 (s, 2H); ^13^C NMR (125 MHz, CDCl_3_) *δ* 166.80, 145.20, 136.13, 134.41, 130.37, 128.93, 128.63, 128.31, 128.14, 117.94, 66.38; MS *m/z*: [M + Mn]^+^ calcd for C_16_H_14_O_2_ 293.28; found 293.2.

### 3.7. Benzyl 1-*H*-pyrrole-2-carboxylate (table 3, entry 7)

^1^H NMR (500 MHz, CDCl_3_) *δ* 8.78 (d, *J* = 4.3 Hz, 1H), 8.16 (d, *J* = 7.8 Hz, 1H), 7.86 (td, *J* = 7.7, 1.4 Hz, 1H), 7.43–7.28 (m, 6H), 5.48 (s, 2H); ^13^C NMR (125 MHz, CDCl_3_) *δ* 164.89, 149.83, 147.85, 137.21, 135.60, 128.63, 128.54, 127.03, 125.34, 67.60; MS *m/z*: [M + H]^+^ calcd for C_13_H_11_O_2_N 214.23; found 214.2.

### 3.8. Benzyl 1-naphthoate (table 3, entry 8)

^1^H NMR (500 MHz, CDCl_3_) *δ* 9.3–9.20 (m, 1H), 8.47–8.33 (m, 1H), 8.07 (d, *J* = 8.2 Hz, 1H), 8.01–7.92 (m, 1H), 7.90–7.27 (m, 9H), 5.62 (s, 2H); ^13^C NMR (125 MHz, CDCl_3_) *δ* 167.34, 136.40, 134.05, 133.72, 131.71, 130.63, 128.85, 128.78, 128.45, 128.04, 127.05, 126.42, 126.07, 124.67, 66.92; HRMS (FT ICR-MS) *m/z*: [M + H]^+^ calcd for C_18_H_14_O_2_ 263.10666; found 263.10599.

### 3.9. Butyl benzoate (table 3, entry 9)

^1^H NMR (500 MHz, CDCl_3_) *δ* 8.07 (d, *J* = 7.5 Hz, 2H), 7.54 (t, *J* = 7.4 Hz, 1H), 7.44 (t, *J* = 7.7 Hz, 2H), 4.34 (t, *J* = 6.6 Hz, 2H), 1.80–1.73 (m, 2H), 1.54–1.46 (m, 2H), 0.99 (t, *J* = 7.4 Hz, 3H); ^13^C NMR (125 MHz, CDCl_3_) *δ* 166.64, 132.76, 130.56, 129.53, 128.30, 64.79, 30.79, 19.28, 13.74; MS *m/z*: [M] calcd for C_11_H_14_O_2_ 178.23; found 178.

### 3.10. Ethyl benzoate (table 3, entry 10)

^1^H NMR (500 MHz, CDCl_3_) *δ* 8.05 (d, *J* = 7.4 Hz, 2H), 7.55 (t, *J* = 7.4 Hz, 1H), 7.43 (t, *J* = 7.7 Hz, 2H), 3.91 (s, 3H); ^13^C NMR (125 MHz, CDCl_3_) *δ* 167.07, 132.89, 131.40, 130.18, 129.56, 128.35, 52.03; MS *m/z*: [M + Na]^+^ calcd for C_8_H_8_O_2_ 136.15; found 136.

### 3.11. Phenethyl cinnamate (table 3, entry 11)

^1^H NMR (500 MHz, CDCl_3_) *δ* 7.72 (d, *J* = 16.0 Hz, 1H), 7.63–7.49 (m, 2H), 7.46–7.34 (m, 5H), 7.33–7.27 (m, 3H), 6.47 (d, *J* = 16.0 Hz, 1H), 4.47 (t, *J* = 7.1 Hz, 2H), 3.13–3.02 (m, 2H); ^13^C NMR (125 MHz, CDCl_3_) *δ* 166.92, 144.89, 137.93, 134.44, 130.33, 128.98, 128.92, 128.57, 128.13, 126.62, 118.10, 65.05, 35.25; MS *m/z*: [M + Na]^+^ calcd for C_17_H_16_O_2_ 275.31; found 275.3.

### 3.12. Benzhydryl cinnamate (table 3, entry 13)

^1^H NMR (500 MHz, CDCl_3_) *δ* 7.80 (d, *J* = 16.0 Hz, 1H), 7.61–7.55 (m, 2H), 7.42–7.33 (m, 11H), 7.32–7.27 (m, 2H), 7.06 (s, 1H), 6.61 (d, *J* = 16.0 Hz, 1H); ^13^C NMR (125 MHz, CDCl_3_) *δ* 166.00, 145.46, 142.23, 140.29, 134.37, 130.43, 128.93, 128.55, 128.40, 128.18, 127.95, 127.44, 127.28, 127.21, 118.04, 80.01; MS *m/z*: [M + Na]^+^ calcd for C_22_H_18_O_2_ 337.38; found 337.3.

### 3.13. Dodecyl benzoate (table 3, entry 14)

^1^H NMR (500 MHz, CDCl_3_) *δ* 8.31 (d, *J* = 8.8 Hz, 2H), 8.23 (d, *J* = 8.8 Hz, 2H), 4.76–4.21 (m, 2H), 1.81 (m, 2H), 1.46 (m, 2H), 1.40–1.25 (m, 16H), 0.90 (t, *J* = 6.8 Hz, 3H); ^13^C NMR (125 MHz, CDCl_3_) *δ* 164.76, 150.48, 135.90, 130.66, 123.52, 66.14, 31.92, 29.64, 29.57, 29.51, 29.35, 29.25, 28.61, 25.99, 22.69, 14.11; MS *m/z*: [M] calcd for C_19_H_29_O_4_N 335.44; found 335.

### 3.14. Phenylmethyl acetate (table 3, entry 15)

^1^H NMR (500 MHz, CDCl_3_) *δ* 7.43–7.38 (m, 4H), 7.38 (s, 1H), 5.15 (s, 2H), 2.11 (s, 3H); ^13^C NMR (125 MHz, CDC_l3_) *δ* 170.84, 136.02, 128.60, 128.29, 66.30, 20.99; MS *m/z*: [M] calcd for C_9_H_10_O_2_ 150.17; found 150.

### 3.15. Benzyl 2,2-diphenylacetate (table 3, entry 16)

^1^H NMR (500 MHz, CDCl_3_) *δ* 7.37–7.27 (m, 15H), 5.22 (s, 2H), 5.11 (s, 1H); ^13^C NMR (125 MHz, CDCl_3_) *δ* 172.32, 138.57, 128.64, 128.59, 128.51, 128.22, 128.17, 127.28, 66.91, 57.06; MS *m/z*: [M + Mn]^+^ calcd for C_21_H_18_O_2_ 357.37; found 357.3.

### 3.16. Benzyl 3-nitrobenzoate (table 3, entry 17)

^1^H NMR (500 MHz, CDCl_3_) *δ* 8.91 (s, 1H), 8.50–8.37 (m, 2H), 7.68 (t, *J* = 8.0 Hz, 1H), 7.44 (ddd, *J* = 18.2, 17.5, 6.4 Hz, 5H), 5.44 (s, 2H); ^13^C NMR (125 MHz, CDCl_3_) *δ* 164.35, 148.33, 135.40, 135.28, 131.95, 129.66, 128.77, 128.67, 128.51, 127.50, 124.70, 67.65; MS *m/z*: [M] calcd for C_14_H_11_O_4_N 257.24; found 257.

**Figure 1.**
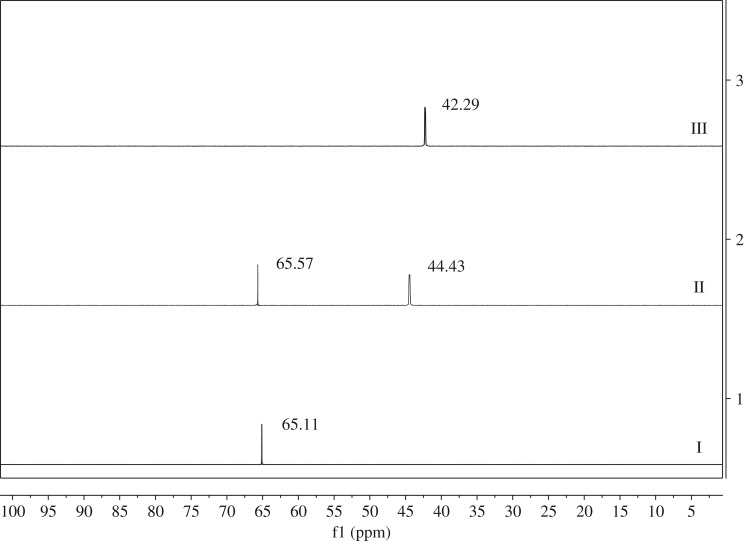
^31^P NMR spectra for the synthesis of RCOOR_1_ (benzyl benzoate). I: TPPO (1 equiv), (COCl)_2_ (1.3 equiv), CH_3_CN (5 ml). II: after addition of RCOOH (benzoic acid, 1 equiv). III: R_1_OH (benzyl alcohol, 1.3 equiv) with Et_3_N (1 equiv) were added to solution II.

**Scheme 4.**
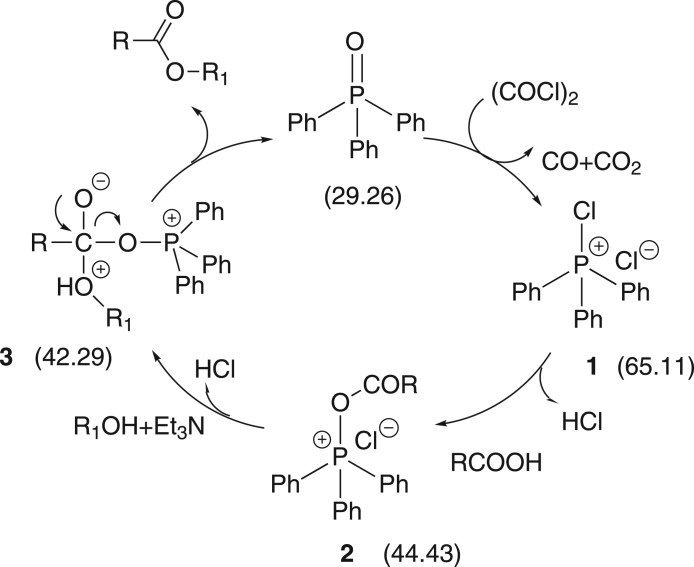
Proposed mechanism of ester synthesis mediated by TPPO and (COCl)_2_.

Based on previous reports [32,47–50], the intermediates of the reaction of TPPO and (COCl)_2_ had been identified as chlorotriphenylphosphonium salt (scheme 4, intermediate 1), which could also be generated in the reaction of phosphine (from the reduction of TPPO) with CCl_4_; we offer a plausible esterification mechanism with the help of ^31^P NMR spectroscopy (figure 1) in scheme 4 for the sake of finding out the role of TPPO with (COCl)_2_ to promote esterification.

Firstly, the solution after adding TPPO (*δ* = 29.26 ppm) [46] with (COCl)_2_ showed a strong singlet at 65.11 ppm (figure 1, I) (*δ* = 65.5 ppm [51]), indicating the formation of intermediate 1. Secondly, after adding benzoic acid, a new singlet was formed at *δ* = 44.43 ppm (figure 1, II) [46], which we hypothesized was due to the formation of an acyl phosphonium salt 2. To exclude the effect of a base, we tested the mixture of TPPO, CH_3_CN with benzoic acid and did not find the analogous singlet; furthermore, we could only see a singlet at 29 ppm. Finally, the salt 2 reacts with alcohol (R_1_OH) to produce corresponding esters and results in a shift of resonance (*δ* = 42.29 ppm) (figure 1, III). To exclude the effect of solvent, we chose equivalent CH_3_CN to replace alcohol; what surprised us was that there was hardly any change in the singlet. Through the post-processing, a sharp singlet of TPPO (*δ* = 29.26 ppm) appeared again. Therefore this particular mechanism needs further study.

## 4. Conclusion

In conclusion, we developed a new and efficient method for the synthesis of esters with excellent yields by the TPPO/(COCl)_2_ system. In comparison with the previous methods for the esterification between carboxylic acids and alcohols, this system offered several advantages. Firstly, this system reduced the side reactions that occurred during the classical Mitsunobu reaction, improved the atom efficiency and reduced the reaction cost, because the raw material TPPO is an industrial by-product of the production of various chemicals and has the characteristic of being widely available, and at the end of this reaction, TPPO could be recycled and only CO, CO_2_ and HCl are wasted. Secondly, the corresponding esters could be generated with excellent yields in mild and neutral conditions, and can be applied to some substrates bearing sensitive groups in contrast to esterification via the formation of acid chloride. Finally, this system has simple experimental operation at the end of the reaction and the reaction liquid was purified by column chromatography directly. Moreover, we also proposed a plausible mechanism according to ^31^P NMR spectroscopy. In our laboratory, we will conduct further investigation of the modified reaction system to extend the application of TPPO.

## Supplementary Material

Electronic Supplementary Material (ESI) for Royal Society Open Science.

## Data accessibility

All data used in this research are included in figures, tables and the electronic supplementary material.
